# Quantitative Structural Analysis of Polystyrene Nanoparticles Using Synchrotron X-ray Scattering and Dynamic Light Scattering

**DOI:** 10.3390/polym12020477

**Published:** 2020-02-19

**Authors:** Jia Chyi Wong, Li Xiang, Kuan Hoon Ngoi, Chin Hua Chia, Kyeong Sik Jin, Moonhor Ree

**Affiliations:** 1 Materials Science Program, School of Applied Physics, Faculty of Science and Technology, Universiti Kebangsaan Malaysia, Bangi 43600, Malaysia; wongjiachyi@gmail.com (J.C.W.); ngoikuanhoon@gmail.com (K.H.N.); 2 Department of Chemistry, Polymer Research Institute, and Pohang Accelerator Laboratory, Pohang University of Science and Technology, Pohang 37673, Korea; lea1990@postech.ac.kr; 3 Pohang Accelerator Laboratory, Pohang University of Science & Technology, Pohang 37673, Korea

**Keywords:** polystyrene nanoparticles, synchrotron X-ray scattering analysis, dynamic light scattering analysis, particle morphology, particle shape, particle size, size distribution, radial density profile

## Abstract

A series of polystyrene nanoparticles (PS-1, PS-2, PS-3, and PS-4) in aqueous solutions were investigated in terms of morphological structure, size, and size distribution. Synchrotron small-angle X-ray scattering analysis (SAXS) was carried out, providing morphology details, size and size distribution on the particles. PS-1, PS-2, and PS-3 were confirmed to behave two-phase (core and shell) spherical shapes, whereas PS-4 exhibited a single-phase spherical shape. They all revealed very narrow unimodal size distributions. The structural parameter details including radial density profile were determined. In addition, the presence of surfactant molecules and their assemblies were detected for all particle solutions, which could originate from their surfactant-assisted emulsion polymerizations. In addition, dynamic light scattering (DLS) analysis was performed, finding only meaningful hydrodynamic size and intensity-weighted mean size information on the individual PS solutions because of the particles’ spherical nature. In contrast, the size distributions were extracted unrealistically too broad, and the volume- and number-weighted mean sizes were too small, therefore inappropriate to describe the particle systems. Furthermore, the DLS analysis could not detect completely the surfactant and their assemblies present in the particle solutions. Overall, the quantitative SAXS analysis confirmed that the individual PS particle systems were successfully prepared with spherical shape in a very narrow unimodal size distribution.

## 1. Introduction

Nanoparticles are accepted widely as materials of several hundred nanometers or less in geometrical dimension. They are commonly thought to have a most simple shape—e.g., spherical form—among nanomaterials. However, they reveal a number of different geometries in detail and a variety of density profiles from the center to the surface. Nevertheless, due to the merits based on nanoscale dimensions, nanoparticles have led to emerging applications in various fields, such as smart coatings, coating additives, polymers, agrochemicals, detergents, lubricants, cutting oils, corrosion inhibitors, catalysts, chemical storage, environmental remediation, energy generation and storage, electro-optics, photonics, microelectronics, cosmetics, pharmaceutics, biosensors, medical diagnostics, medical therapy, drug delivery carriers, foods, and so on [1,2,3,4,5,6,7,8,9,10,11,12,13,14].

For a material, all properties are generally known to depend upon the morphological structure and molecular characteristics. In the case of nanoparticles, the properties and performance are also highly dependent upon the morphology details as well as the chemical characteristics [1,7,8,9,10,11,12,13,14]. The morphology details include three-dimensional geometry, surface texture, size and size distribution, surface-to-volume ratio, and density gradient profile. In particular, particle size is known as a crucial factor to govern the properties and performance [13,14,15,16,17,18,19,20]. To measure nanoparticle size, dynamic light scattering is currently used most widely because of the experimental simplicity and automatized data analysis scheme using the instruments developed in compact type [21,22,23,24,25,26]. However, dynamic light scattering very often provides unrealistically large size and distribution [27,28,29,30,31,32,33,34]. Electron microscopy is also widely used to characterize nanoparticles, but always requires specific measurement conditions such as dried or freeze samples and vacuum environment [35,36,37]. As an alternative of electron microscopy, atomic force microscopic analysis is applicable for nanoparticles in aqueous medium or another solvent medium [28,38,39,40,41]. There are several other methods available, including X-ray scattering, static light scattering, laser diffraction, neutron scattering, fluorescence correlation spectroscopy, sedimentation, sieving, optical particle counting, electrozone sensing, and resistive pulse sensing [42,43,44,45,46,47,48,49,50,51,52,53,54,55,56]. However, the individual methods have some disadvantages including detection limit and valid boundary condition, leading to differences even in the size of nanoparticle [28,32,33,40,43,49,50,53,54,55,56]. Furthermore, they often face difficulties in analyzing measured data mathematically in a quantitative manner. The correct interpretation and understanding of analysis results in could also be challenging because of the different underlying metrological theories, concepts, and boundary conditions. Therefore, nanoparticles still need urgent advances even in the size and size distribution measurement with higher precision and higher accuracy. Their structural details (geometrical shape, surface texture, density gradient profile, etc.) are further analyzed for better understanding and appropriate utilization. 

In this study, we have attempted to investigate a series of polystyrene (PS) nanoparticles in various sizes in order to get insights into their morphological structure and size distribution. For this investigation, synchrotron small-angle X-ray scattering (SAXS) technique has been chosen because of its ability to determine structure and size details and the availability of synchrotron radiation X-ray source and experimental beamline facility. Dynamic light scattering (DLS) method has been additionally chosen due to its availability in an economic way as a compact and handy instrument and the easy usage with a built-in automatized data analysis software package. The quantitative SAXS analysis provides geometrical shape, size, size distribution, and density gradient profile. The DLS gives additional information on the particle size and distribution.

## 2. Materials and Methods

A series of PS nanoparticles were received from Thermo Fisher Scientific Company (Seoul, Korea): PS-1 (Nanosphere™ 3080A, 1 wt % in aqueous solution), PS-2 (Nanosphere™ 3050A, 1 wt % in aqueous solution), PS-3 (Nanosphere™ 3030A, 1 wt % in aqueous solution), and PS-4 (Nanosphere™ 3020A, 1 wt % solid in aqueous solution). For each sample solution, a part was taken out, put into a centrifuge tube, and followed by centrifugation at 12,000 rpm for 32 h in a refrigerated centrifuge (model 1248R, LABOGENE, Seoul, Korea) at 25 °C. Then, the upper liquid layer was carefully pipetted out from the centrifuge tube without touching the sedimented nanoparticles, put into a vial, and kept as a supernatant for X-ray scattering measurements. 

X-ray scattering measurements were conducted at the 4C beamline [32,44,45,57] of the PLS-II facility (third-generation synchrotron facility of 3.0 GeV power), Pohang Accelerator Laboratory (Pohang, Korea). The X-ray beam with a wavelength λ of 0.07336 nm was used. A two-dimensional (2D) charged-coupled detector (CCD: model Rayonix 2D SX 165, Evanston, IL, USA) was employed. Quartz capillary cells with 1.5 mm outer diameter were used. The sample-to-detector distances (SDD) of 1.0 m and 4.0 m were chosen. The data collection time was 30 s. The scattering angles were calibrated by using a precalibrated Ti-SBA-15 (Sigma-Aldrich Company, Seoul, Korea) and silver behenate (Tokyo Chemical Industry (TCI), Tokyo, Japan) as standards. The individual 2D scattering data were circularly averaged with respect to the X-ray beam center and then followed by normalizing to the transmitted X-ray beam intensity which was monitored with a scintillation counter positioned behind the sample. The scattering data was further corrected for the scattering arising from either the water or the supernatant.

DLS measurements were carried out at 25.0 °C (±0.1 °C) by using a Malvern DLS instrument (model Zetasizer Nano ZS90, Malvern Instruments Ltd., Worcestershire, UK) equipped with a He-Ne laser source of 632.8 nm wavelength; a detector was set at a scattering angle of 90°. Low-volume quartz batch cuvettes (model ZEN2112, Malvern Instruments Ltd., Worcestershire, UK) were employed as sample cells. 

## 3. Results and Discussion

### 3.1. PS-1

**Figure 1:**
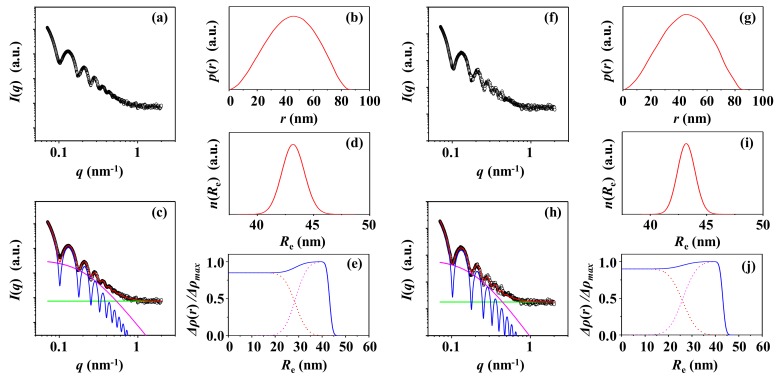
X-ray scattering analysis of PS-1. (**a**) SAXS profile measured at room temperature and corrected for water as the solution medium: (**b**) pair distance distribution functions *p*(*r*) obtained from the scattering profile using the IFT method; (**c**) data analysis results; (**d**) radius distribution obtained by the data analysis in (**c**); (**e**) density distribution obtained by the data analysis in (**c**). (**f**) SAXS profile measured at room temperature and corrected for the supernatant as a solution medium: (**g**) pair distance distribution functions *p*(*r*) obtained from the scattering profile using the IFT method; (**h**) data analysis results; (**i**) radius distribution obtained by the data analysis in (**h**); (**j**) density distribution obtained by the data analysis in (**h**). (**c**, **h**) the open symbols are the measured data and the red solid line represents the sum of the profiles obtained by fitting the data using two-phase ellipsoid model (blue line) and local random two-phase contributions (purple line) and the background (green line).

Figure 1a presents a representative of the synchrotron X-ray scattering data of the PS-1 particle in aqueous solution measured at room temperature and corrected for the water medium. The scattering profile has been attempted to be analyzed in a qualitative manner by using the indirect Fourier transformation (IFT) analysis [58], which is a model independent scattering analysis method; the IFT method detail is given in Supporting Information. The obtained *p*(*r*) profile is shown in Figure 1b, providing structural information: (i) *R*_g,IFT_ = 33.8 nm (radius of gyration), (ii) *R*_max,IFT_ = 45.5 nm (radius at the peak maximum), and (iii) *D*_max,IFT_ = 87.5 nm (maximum dimension) (Table 1). The *p*(*r*) profile is apparently close to a bell curve that is commonly observed for an ideal sphere. *R*_max,IFT_*/R*_g,IFT_ = 1.35, which is close to 1.36 for an ideally homogenous sphere [4,59]. *D*_max,IFT_*/R*_max,IFT_ = 1.92, which is close to 2 for an ideal sphere. These results collectively inform that PS-1 is a sphere-like nanoparticle revealing a certain size, namely *R*_g,IFT_ = 33.8 nm and *R*_max,IFT_ = 45.5 nm. 

With the IFT analysis results, the X-ray scattering data have been further subjected to quantitative structural model analysis. Various structural models have been considered and tested. As a result, a two-phase ellipsoidal model approach is found most suitable. As shown in Figure 1c, the scattering data are well fitted by using the two-phase ellipsoidal model combined with the local density fluctuations based on random two phases (see Figure 1c); the scattering formulas are given in Supporting Information. This analysis provides the nanoparticle shape and structural parameter details (Table 1). The ellipsoidicity ratio *ε* [= (polar radius)/(equatorial radius)] is determined to be 1.0, confirming that PS-1 is spherical. The radius of gyration *R*_g_ is 33.5 nm, which is very close to that determined by the IFT analysis. The mean radius *R*_e_ is 43.2 nm, which consists of a core radius *r*_c_ of 29.2 nm, and a shell thickness *t*_s_ of 14.0 nm. In the whole particle, the average correlation length *ξ* of local density fluctuations (i.e., random two phases) is additionally estimated to be 4.6 nm. These local density fluctuations may originate mainly from the chemical crosslinks formed in the PS-1 synthesized by using emulsion polymerization technique. The radius (i.e., size) distribution of PS-1 particles is found to follow the Schultz–Zimm function [60]; the obtained radius distribution is presented in Figure 1d. 

The radial electron density distribution profile Δ*ρ*(*r*) is one of the interesting structural parameters for understanding the PS-1 particle. Thus, the density profile has been first attempted to be extracted from the *p*(r) profile obtained from the IFT analysis. However, this extraction approach could give unrealistic density profiles as multiple solutions. This failure may be attributed to the IFT software’s limitations including the assumption of monodispersity in size distribution. Instead, the numerical Fourier transformation of the respective scattering amplitudes determined in the quantitative structural model analysis is found to provide a more realistic density profile, as shown in Figure 1e; the analysis detail is in Supporting Information. 

This quantitative analysis has been extended to extract *p*(*r*) profile. Namely, with the structural parameters determined by the quantitative model analysis, a scattering profile has been reconstructed and then Fourier-transformed, giving *p*(*r*) profile (Figure S1 in Supplementary Materials). From the *p*(*r*) profile, a set of structural parameters (*R*_g_, *R*_max_, and *D*_max_) has been obtained, as listed in Table S1. Overall, the extracted *p*(*r*) profile and resulting structural parameters are reasonably well matched with those determined directly from the measured scattering data by the IFT analysis. This crosscheck again confirms the validity of the density profile extracted above and furthermore the scattering data analysis done successfully.

It was informed from the supplier that the aqueous PS-1 solution includes a small quantity of surfactant which was employed in the particle preparation by surfactant-based emulsion polymerization; such surfactant is known to further stabilize the dispersion of the resulting particles in the solution. The surfactant residue may also cause scattering with X-ray beam and influence the X-ray scattering data arising for the PS-1 solution. Thus, the PS-1 solution has been separated into the PS-1 particles and the supernatant by centrifugation. For the obtained supernatant, X-ray scattering measurement has been carried out and then used to correct the X-ray scattering data measured for the PS-1 solution. The corrected scattering data are presented in Figure 1f and then followed by IFT and structural model analyses. The analysis results are shown in Figure 1h–j. The obtained structural parameters are almost the same as those determined by analyzing the X-ray scattering data corrected with the water medium (Table 1). These analyses collectively confirm that the presence of the surfactant molecules in the solution causes no positive or negative contributions on the X-ray scattering data of the PS-1 solution. This result may be attributed to large size differences between the PS-1 particles and the surfactant molecules including their possible assemblies and possibly low concentration of the surfactant in the solution. 

**Figure 2:**
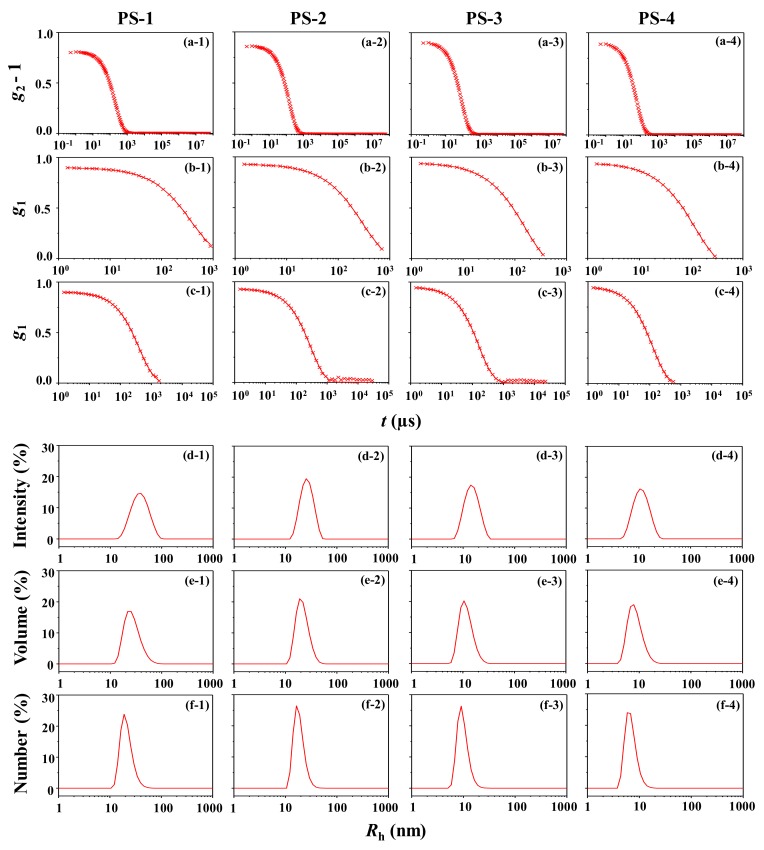
DLS analyses of PS nanoparticles at 25 °C: (**a-1** to **a-4**) autocorrelation profiles measured; (**b-1** to **b-4**) data analysis results, where the symbols are the measured data and the red solid lines were obtained by fitting the data using the cumulant method in the Zetasizer program package; (**c-1** to **c-4**) data analysis results, where the symbols are the measured data and the red solid lines were obtained by fitting the data using the non-negatively constrained least square (NNLS) deconvolution algorithm included in the Zetasizer program package; (**d**-1 to **d-4**) intensity-weighted radius distributions obtained by the data analyses; (**e**) volume-weighted radius distributions obtained from the radius distributions in (**d-1** to **d-4**) and (**f**) number-weighted radius distributions obtained from the radius distributions in **(e-1** to **e-4**).

The PS-1 solution has been additionally examined by DLS. Figure 2a-1 shows a representative second order autocorrelation function *g*_2_(*q*,*t*) constructed from the fluctuating scattered intensity *I*_s_(*q*,*t*) data measured at a fixed scattering angle of 90° as a function of time. From the *g*_2_(*q*,*t*) profile, the first order autocorrelation function *g*_1_(*q*,*t*) (i.e., field autocorrelation function) has been extracted by using the Siegert relation [32,62,63] and then analyzed using the Malvern Zetasizer analysis software package based on the cumulant method and the non-negatively constrained least square (NNLS) deconvolution algorithm; the DLS data analysis details are given in Supporting Materials. Although the Cumulant algorithm always assumes monodispersed size distribution, interestingly it fits the *g*_1_(*q*,*t*) profile (Figure 2b-1), providing information on the hydrodynamic radius and polydispersity of the particle. The *g*_1_(*q*,*t*) profile is further fitted in a quantitative manner by the NNLS algorithm, giving information on the mean particle size and distribution (Figure 2c-1,d-1 and f-1). The DLS analysis results are summarized in Table 1. 

The cumulant-based analysis gives a polydispersity *PDI*_DLS_ of 0.154. Taking into account the upper limit (*PDI*_DLS_ = 0.7) of quality DLS data analysis suggested as a guideline [26], such small *PDI_DLS_* value is an indication that the DLS data analysis has been done in a reasonably good quality manner. In addition, the NNLS deconvolution analysis provides a single radius distribution peak. These results collectively inform that PS-1 has a unimodal size distribution. The hydrodynamic radius *R*_h,z_ is 33.5 nm, which is a *z*-averaged radius of gyration of the particle. The mean radius *R*_h_ is extracted in three different modes: *R*_h,intensity_ = 39.9 nm with a standard deviation *σ* of 14.7 nm, which is estimated from the intensity-weighted radius distribution (Figure 2d-1), *R*_h,volume_ = 28.0 nm with *σ* = 10.5 nm, obtained from the volume-weighted radius distribution (Figure 2e-1), and *R*_h,number_ = 21.6 nm with *σ* = 6.3 nm, determined from the number-weighted radius distribution (Figure 2f-1).

**Figure 3:**
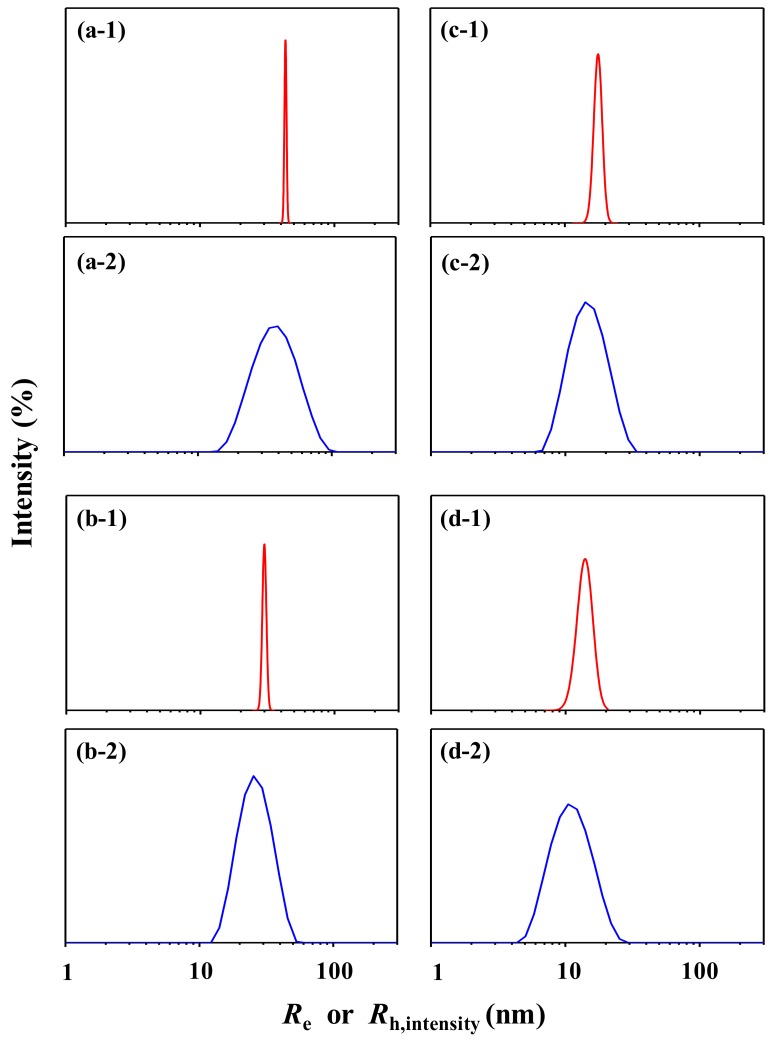
Radius distributions of PS particles determined by X-ray scattering and DLS analyses: (**a-1**) PS-1 (*σ* = 0.8 nm), from X-ray scattering; (**a-2**) PS-1 (*σ* = 14.7 nm), from DLS; (**b-1**) PS-2 (*σ* = 1.0 nm), from X-ray scattering; (**b-2**) PS-2 (*σ* = 7.4 nm), from DLS; (**c-1**) PS-3 (*σ* = 1.3 nm), from X-ray scattering; (**c-2**) PS-3 (*σ* = 4.7 nm), from DLS; (**d-1**) PS-4 (*σ* = 1.8 nm), from X-ray scattering; (**d-2**) PS-4 (*σ* = 3.9 nm), from DLS. Here, it is noted that the radius distributions based on the scattering intensities in (**a-1**), (**b-1**), (**c-1**), and (**d-1**) were obtained from the radius distribution based on the number populations in Figure 1i, Figure 4i, Figure 5i, and Figure 6i using a relation of the scattering intensity and the volume of the particles in population; such relation is given in Supporting Information. The X-ray scattering analyses were conducted for the scattering data corrected with the supernatant media.

Interestingly, the *R*_h,z_ value is in good agreement with the *R*_g_ determined by the quantitative X-ray scattering analysis. Furthermore, the *R*_h,intensity_ value is close to the mean radius (43.2 nm = *R*_e_) determined by X-ray scattering analysis and much closer to that (40.5 nm) estimated by transmission electron microscopy (TEM) analysis [61]. However, the *R*_h,volume_ and *R*_h,number_ are significantly smaller than those determined by X-ray scattering and TEM analyses; they are 31–35% and 47–50% smaller than those determined by X-ray scattering and TEM analyses, respectively. Moreover, the radius distributions are unrealistically 4.2 to 14.7 times larger than those (1.0 and 1.5 nm) determined by X-ray scattering and TEM analyses. Overall, the DLS analysis provides valid *R*_h,z_ and *R*_h,intensity_ values but unrealistically broad size distribution (Figure 3a-1,a-2). The *R*_h,volume_ and *R*_h,number_ are unrealistic and therefore impractical. These unpleasant outputs are attributed to the high uncertainties in the deconvolution of autocorrelation function and very limited resolution of the DLS instrument which may originate from several factors such as (i) much longer wavelength of the used laser source compared to the particle size; (ii) very compact optic geometry; and (iii) only one fixed scattering angle of the detector. 

### 3.2. PS-2

**Figure 4:**
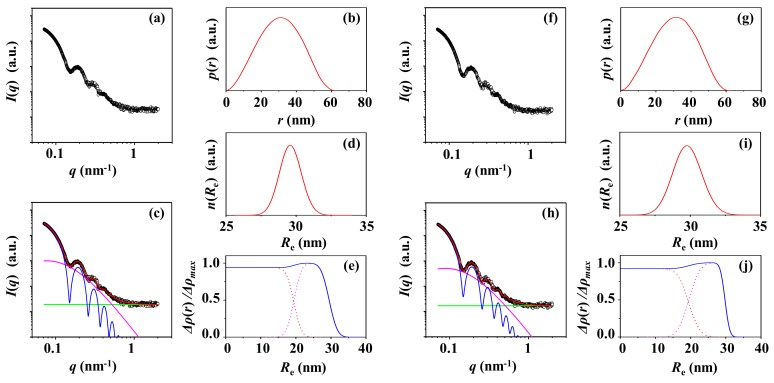
X-ray scattering data analysis of PS-2 particle. (**a**) SAXS profile measured at room temperature and corrected for water as the solution medium: (**b**) pair distance distribution functions *p*(*r*) obtained from the scattering profile using the IFT method; (**c**) data analysis results; (**d**) radius distribution obtained by the data analysis in (**c**); (**e**) density distribution obtained by the data analysis in (**c**). (**f**) SAXS profile measured at room temperature and corrected for the supernatant as a solution medium: (**g**) pair distance distribution functions *p*(*r*) obtained from the scattering profile using the IFT method; (**h**) data analysis results; (**i**) radius distribution obtained by the data analysis in (**h**); (**j**) density distribution obtained by the data analysis in (**h**). (**c**, **h**) the open symbols are the measured data and the red solid line represents the sum of the profiles obtained by fitting the data using two-phase ellipsoid model (blue line) and local random two-phase contributions (purple line) and the background (green line).

Figure 4a shows a representative of the synchrotron X-ray scattering data of the PS-2 solution corrected for the water medium. Figure 4f presents the X-ray scattering data of the PS-2 solution corrected for the supernatant. These scattering data have been successfully analyzed by using both the IFT method and the two-phase ellipsoidal model approach combined with the local density fluctuations based on random two phases. The analysis results are illustrated in Figure 4b–e,g–j. The determined structural parameters are listed in Table 1. 

From the scattering data corrected with the water medium, the *p*(r) profile is obtained to be like symmetric bell shape with *D*_max,IFT_*/R*_max,IFT_ = 2.04 and *R*_max,IFT_*/R*_g,IFT_ = 1.33, which is close to 1.36. Together with *R*_g_*/R*_g,IFT_ = 0.98 and *ε* = 1.0, these results collectively confirm that PS-2 is spherical. The spherical PS-2 particle is characteristic of a set of structural parameters: *R*_g_ = 22.9 nm, *R*_e_ = 29.6 nm, *r*_c_ = 19.6 nm, *t*_s_ = 10.0 nm, and *ξ* = 5.4 nm. The Δ*ρ*(*r*) profile has been extracted. Similar shape and structural parameters have been determined from the scattering data corrected for the supernatant, informing that the X-ray scattering data of the PS-2 solution are influenced very little by the surfactant molecules present in the solution. 

The DLS data of the PS-2 solution are presented in Figure 2a-2. The *g*_1_(*q*,*t*) profile is reasonably fitted by the cumulant method as well as by the NNLS algorithm (Figure 2b-2,c-2). The DLS analysis results are summarized in Table 1 and Figure 2d-2–f-2. *PDI*_DLS_ = 0.055, which is lower than that of PS-1 and much lower than the upper limit (0.7) of quality DLS data analysis. However, *R*_h,z_ = 25.0 nm, that is 9% larger than the *R*_g_ determined by the quantitative X-ray scattering analysis. *R*_h,intensity_ = 26.9 nm, which is 9% smaller than the *R*_e_ determined by X-ray scattering analysis, but 5% larger than the mean radius (25.5 nm) determined by TEM [61]. *R*_h,volume_ = 21.9 nm and *R*_h,number_ = 18.6 nm, which are much smaller than the mean radii determined by X-ray scattering and TEM analyses. Depending on the weighted modes (namely, intensity-, volume-, and number-weighted distributions), the *σ* values range in 7.4 to 4.5, which are much larger than those (0.8 and 1.5) determined by X-ray scattering and TEM analyses respectively. The radius distributions are unrealistically too broad (Figure 3b-1,b-2). Overall, only *R*_h,z_ and *R*_h,intensity_ values are meaningful for PS-2. 

### 3.3. PS-3

**Figure 5:**
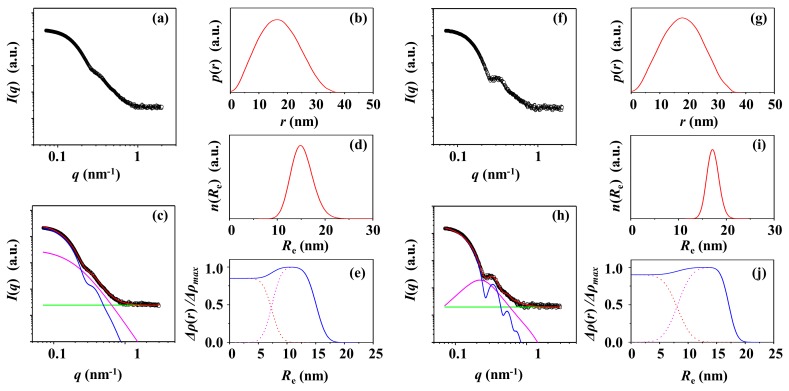
X-ray scattering data analysis of PS-3 particle. (**a**) SAXS profile measured at room temperature and corrected for water as the solution medium: (**b**) pair distance distribution functions *p*(*r*) obtained from the scattering profile using the IFT method; (**c**) data analysis results; (**d**) radius distribution obtained by the data analysis in (**c**); (**e**) density distribution obtained by the data analysis in (**c**). (**f**) SAXS profile measured at room temperature and corrected for the supernatant as a solution medium: (**g**) pair distance distribution functions *p*(*r*) obtained from the scattering profile using the IFT method; (**h**) data analysis results; (**i**) radius distribution obtained by the data analysis in (**h**); (**j**) density distribution obtained by the data analysis in (**h**). (**c**, **h**) the open symbols are the measured data and the red solid line represents the sum of the profiles obtained by fitting the data using two-phase ellipsoid model (blue line) and local random two-phase contributions (purple line) and the background (green line).

Figure 5a shows a representative of the X-ray scattering data of the PS-3 solution corrected for the water medium. The *p*(*r*) profile is slightly asymmetric; in particular, the part in the high *r* region is a little bit far from the symmetry (Figure 5b). From the IFT analysis, *R*_g,IFT_ = 12.9 nm and *R*_max,IFT_ = 16.3 nm. *R*_max,IFT_*/R*_g,IFT_ = 1.26, which is somewhat smaller than 1.36 for an ideally homogenous sphere. *D*_max,IFT_*/R*_max,IFT_ = 2.33, which is larger than 2 for an ideal sphere. 

The scattering data have been further analyzed in a quantitative manner. The scattering profile is satisfactorily fitted by using the two-phase ellipsoidal model combined with the local density fluctuations based on random two phases, as shown in Figure 5c. The quantitative analysis results are compared with the IFT analysis results in Table 1. *R*_g_ = 11.2 nm, which is 13% smaller than that determined by the IFT analysis. Other structural parameters obtained are as follows: *R*_e_ = 14.5 nm, *r*_c_ = 7.3 nm, *t*_s_ = 7.2 nm, *ξ* = 5.0 nm, and *ε* = 1.0. In addition, the density profile is determined (Figure 5e). In particular, the *ε* value indicates that PS-3 is spherical. PS-3 is found to have a unimodal radius distribution (Figure 5d).

Moreover, the scattering profile has been corrected for the supernatant medium, which is a little bit different from that corrected for the water medium. The scattering data have been analyzed successfully by using the IFT and structural model approaches. The corrected scattering data and analysis results are displayed in Figure 5f–j. *R*_max,IFT_*/R*_g,IFT_ = 1.33 (= 18.0/13.5), which is much closer to 1.36; *D*_max,IFT_*/R*_max,IFT_ = 2.13 (= 38.4/18.0), which is close to 2.0. These ratios suggest that the shape of PS-3 is closer to spherical. From the structural model analysis, *R*_g_ = 12.9 nm, *R*_e_ = 16.5 nm, *r*_c_ = 8.3 nm, *t*_s_ = 8.2 nm, *ξ* = 2.9 nm, and *ε* = 1.0. The density profile is determined additionally. Overall, it is again vindicated that PS-3 is spherical and in a unimodal radius distribution.

The DLS data and analysis results are shown in Figure 2a-3–f-3) and Table 1. Briefly, the *PDI*_DLS_ = 0.085, *R*_h,z_ = 14.0 nm, *R*_h,intensity_ = 15.3 nm, *R*_h,volume_ = 11.9 nm, and *R*_h,number_ = 9.9 nm. A unimodal radius distribution is found.

The above X-ray scattering and DLS analyses collectively provide key structural features on the PS-3 particle as follows. 

First, the X-ray scattering profile corrected for the water medium is slightly different from that corrected for the supernatant medium. This difference is indicative of discernible contributions of the surfactant molecules and their possible assemblies present in the PS-3 solution to the measured X-ray scattering signals.

Second, for the X-ray scattering data corrected from the water medium, the *p*(*r*) profile is somewhat asymmetric particularly in the high *r* region. As a result, the *R*_max,IFT_*/R*_g,IFT_ and *D*_max,IFT_*/R*_max,IFT_ values are significantly far from those of a spherical particle. In comparison, for the X-ray scattering data corrected from the supernatant medium, the *p*(*r*) profile is more like symmetric; the *R*_max,IFT_*/R*_g,IFT_ and *D*_max,IFT_*/R*_max,IFT_ values are close to those of a spherical particle. The structural parameters (*R*_g,IFT_, *R*_g_, and *R*_e_) are larger than those determined from the scattering data corrected for the water medium. The radius distribution is much narrower than that obtained from the scattering data corrected for the water medium. These structural differences could be attributed to the surfactant molecules and their possible assemblies present in the PS-3 solution. Namely, in the case of PS-3, the particle dimension (*R*_g_ = 12.9 nm and *R*_e_ = 16.5 nm) is not large enough to override all scattering signals from the surfactant molecules and their possible assemblies present together in the solution. As discussed above, these kinds of surfactant effects apparently could not be observed for the PS-1 (*R*_g_ = 33.5 nm and *R*_e_ = 43.2 nm) and PS-2 (*R*_g_ = 23.1 nm and *R*_e_ = 29.8 nm) solutions. 

Third, the *R*_h,z_, which is determined by the DLS analysis, is 25% larger than the *R*_g_ determined from the scattering data corrected for the water medium and only 9% larger than the *R*_g_ determined from the scattering data corrected for the supernatant medium. The *R*_h,intensity_ value is 7% larger than *R*_e_ determined from the scattering data corrected for the water medium and 6% smaller than that determined from the scattering data corrected for the supernatant medium. These results collectively suggest that the DLS data are mainly driven from the PS-3 particles but negligibly contributed from the surfactant molecules and their possible assemblies present in the solution. Nevertheless, the particle radius distribution is much broader than that determined by the X-ray scattering analysis. Such the insensitivity to the surfactant molecules as well as the broader radius distribution are caused by the laser source’ long wavelength, compact optic geometry, and 90°-fixed scattering angle in the DLS instrument.

Finally, the *R*_h,volume_ and *R*_h,number_ values are much smaller than the *R*_h,intensity_ value as well as the *R*_e_ value determined by the X-ray scattering analysis. All radius distributions (intensity-, volume-, and number-weighted size distributions) are too broad unrealistically, compared to that determined by the X-ray scattering analysis (Figure 3c-1,c-2). 

### 3.4. PS-4

**Figure 6:**
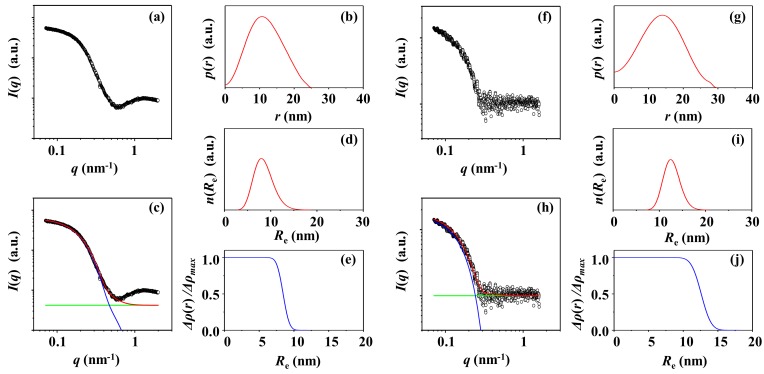
X-ray scattering data analysis of PS-4 particle. (**a**) SAXS profile measured at room temperature and corrected for water as the solution medium: (**b**) pair distance distribution functions *p*(*r*) obtained from the scattering profile using the IFT method; (**c**) data analysis results; (**d**) radius distribution obtained by the data analysis in (**c**); (**e**) density distribution obtained by the data analysis in (**c**). (**f**) SAXS profile measured at room temperature and corrected for the supernatant as a solution medium: (**g**) pair distance distribution functions *p*(*r*) obtained from the scattering profile using the IFT method; (**h**) data analysis results; (**i**) radius distribution obtained by the data analysis in (**h**); (**j**) density distribution obtained by the data analysis in (**h**). (**c**, **h**) the open symbols are the measured data and the red solid line represents the sum of the profiles obtained by fitting the data using two-phase ellipsoid model (blue line) and local random two-phase contributions (purple line) and the background (green line).

Figure 6a shows a representative X-ray scattering profile of the PS-4 solution corrected for the water medium. The IFT analysis provides an asymmetric *p*(*r*) profile (Figure 6b), *R*_g,IFT_ = 8.9 nm, *R*_max,IFT_ = 10.7 nm, and *D*_max,IFT_ = 25.4 nm (Table 1). *R*_max,IFT_*/R*_g,IFT_ = 1.20 and *D*_max,IFT_*/R*_max,IFT_ = 2.37, informing that PS-4 behaves a shape far from sphere. The scattering data have been further analyzed in a quantitative manner. The scattering data in the region of *q* < 0.6 nm^-1^ are well fitted by using the two phase ellipsoidal model combined with the local density fluctuations based on random two phases; but the scattering data in the region of *q* > 0.6 nm^-1^ could be unfitted completely (Figure 6c). From the fitted scattering data part, structural parameters have been extracted: *R*_g_ = 6.6 nm, *R*_e_ = 8.5 nm, *r*_c_ = 8.5 nm, *ε* = 1.0, unimodal size distribution, and density profile (Table 1; Figure 6d,e).

Figure 6f presents the X-ray scattering data corrected for the supernatant medium. This scattering profile does show no peak over the region of *q* > 0.6 nm^−1^, which is different from that corrected for the water medium. The scattering profile has been analyzed by using the IFT method and the two-phase ellipsoidal model combined with the local density fluctuations based on random two phases (Figure 6g,h). The IFT analysis gives an inadequate *p*(*r*) profile in the low r region which represents the smaller structural details. It is probably due to the noisy scattering data in the high q region. The structural parameters were obtained: *R*_g,IFT_ = 10.4 nm, *R*_max,IFT_ = 14.1 nm, *D*_max,IFT_ = 30.0 nm, *R*_max,IFT_*/R*_g,IFT_ = 1.36, and *D*_max,IFT_*/R*_max,IFT_ = 2.13. The model analysis provides density profile, radius distribution, and a set of parameters: *R*_g_ = 9.8 nm, *R*_e_ = 12.7 nm, and *ε* = 1.0 (Figure 6i,j and Table 1). Furthermore, the numerical Fourier transformation was performed on the extrapolated scattering intensity profile from the structural model analysis to obtain the *p*(*r*) profiles. All *p*(*r*) profiles that were extracted using this method are shown in Supporting Information. The *R*_g_ values extracted from the IFT analysis and numerical Fourier transformation are similar.

In addition, the DLS analysis gives radius and radius distributions: *R*_h,z_ = 10.4 nm, *PDI*_DLS_ = 0.093, *R*_h,intensity_ = 11.6 nm, *R*_h,volume_ = 8.6 nm, and *R*_h,number_ = 7.0 nm (Figure 2a-4–f-4). This analysis confirms again that PS-4 exhibits a unimodal radius distribution.

The above X-ray scattering and DLS analyses provide structural feature details on PS-4 below.

First, surprisingly the PS-4 solution shows a broad and weak peak over the region of *q* > 0.6 nm^−1^ in the X-ray scattering profile corrected for the water medium, in addition to the scattering signals originated from the PS-4 particle itself. Such broad and weak peak could not be discernible in the X-ray scattering profile corrected for the supernatant medium. For the scattering peak centered at *q* = 1.3 nm^−1^, the *d*-spacing is estimated to be 4.8 nm. This *d*-spacing value is much larger than the dimension of the individual surfactant molecules but smaller than the dimension of the PS-4 particle. Taking these facts into account, the scattering peak at *q* > 0.6 nm^−1^ may be attributed to assemblies of the surfactant molecules present in the PS-4 solution. Their size is not small enough, compared to the dimension of PS-4. Therefore, their contributions are severely reflected in the measured scattering profile. 

Second, the structural model analysis confirms that PS-4 is a single-phase spherical particle rather than a two-phase particle. This result may be an evidence that the PS-4 particles were prepared in a relatively small scale and thus could not have enough opportunity to be grown as two-phase particle during their emulsion polymerization process. 

Third, for the X-ray scattering profile corrected with the water medium, its *p*(*r*) profile shows asymmetric characteristics. Such characteristics are directly reflected in *R*_max,IFT_*/R*_g,IFT_ and *D*_max,IFT_*/R*_max,IFT_; these ratios are deviated from 1.36 and 2.00, respectively. These results collectively support the presence of the surfactant molecules and their assemblies in the PS-4 solution. 

Fourth, the *R*_h,z_ obtained by the DLS analysis is 58% larger than the *R*_g_ determined from the scattering data corrected for the water medium but only 6% larger than the *R*_g_ determined from the scattering data corrected for the supernatant medium. The *R*_h,intensity_ value is 36% larger than the *R*_e_ determined from the scattering data corrected for the water medium. However, the value is 9% smaller than that determined from the scattering data corrected for the supernatant medium. These results confirm again that the surfactant molecules employed in the PS-4 particle synthesis remain in the particle suspension. Moreover, these comparisons suggest that the DLS data are mainly driven from the PS-4 particles, but surprisingly insensitive even to the presence of the surfactant assemblies revealing a *d*-spacing of 4.9 nm in the X-ray scattering in addition to the surfactant molecules. Such insensitivities to the existing surfactants and their assemblies are evidence for the poor limits of the DLS instrument, consequently causing errors in determining particle size and distribution. 

Finally, the *R*_h,volume_ and *R*_h,number_ values are much smaller than the *R*_h,intensity_ value as well as the *R*_e_ value determined by the X-ray scattering analysis. Furthermore, the radius distributions estimated by the DLS analysis are always too broad, compared to that determined by the X-ray scattering analysis (Figure 3d-1,d-2). Therefore, the *R*_h,volume_, *R*_h,number_ and radius distributions provided by the DLS analysis are meaningless practically. 

## 4. Conclusions

In this study, a series of PS nanoparticles, which were prepared by conventional surfactant-assisted emulsion polymerization, has been investigated in terms of morphological structure and size distribution.

The quantitative X-ray scattering analysis has been successfully performed on the PS particles in aqueous media, providing key features as follows. The PS solution individuals are found of (i) spherical particles in very narrow unimodal size distribution and (ii) surfactant molecules and their assemblies. The PS-1, PS-2, and PS-3 particle individuals are characteristic of revealing two-phase (core and shell) nanoparticles, whereas PS-4 is a single-phase nanoparticle; they all exhibit unique electron density profiles. For the PS particle individuals, structural parameter details have been determined in high precision and accuracy. The particle size (*R*_g_ and *R*_e_) is in the decreasing order: PS-1 > PS-2 > PS-3 > PS-4; in contrast, the broadening of size distribution is in the increasing order: PS-1 < PS-2 < PS-3 < PS-4. 

The PS solutions have been further examined by DLS analysis. All analyses have done with very low *PDI*_DLS_ values (0.055–0.154), suggesting that all PS particles are spherical. Fortunately, due to the spherical nature, the particle size (*R*_h_ and *R*_h,intensity_) of each particle system is determined to be reasonably close to those (*R*_g_ and *R*_e_) extracted from the X-ray scattering and TEM analysis. All PS particle systems are confirmed to behave unimodal size distributions. Nevertheless, the determined size distributions are too broad unrealistically, compared to those extracted by X-ray scattering analysis. In contrast, the *R*_h,volume_ and *R*_h,number_ values are always too small unrealistically. Furthermore, the DLS analysis are completely insensitive to the surfactant molecules and their assemblies present in the particle solutions. Overall, the compact and fixed-angle DLS instrument of this study is confirmed to have severe resolution limits and thus applicable to measure *R*_h_ and *R*_h,intensity_ for only spherical particles; the *R*_h,volume_, *R*_h,number_, and all different mode based size distributions could give no meaningful information even though they were extracted from the spherical particles.

In summary, the quantitative X-ray scattering analysis together with qualitative DLS analysis has confirmed that the individual PS particle systems were successfully prepared with spherical shape in a very narrow unimodal size distribution. The X-ray scattering analysis has further provided morphological structure details (shape, dimension, density profile, size, and size distribution) of all PS nanoparticles.

## Figures and Tables

**Table 1:** Structural parameters of PS nanoparticles obtained by SAXS, DLS, and TEM analyses.

PS Nanoparticles								
**Structure Parameter**	PS-1		PS-2		PS-3		PS-4	
	Water *^a^*	Supernatant *^b^*	Water	Supernatant	Water	Supernatant	Water	Supernatant
*SAXS Analysis*								
*R_e_^c^*(nm)	43.2 (1.0) *^d^*	43.2 (0.8)	29.6 (0.8)	29.8 (1.0)	14.5 (2.5)	16.5 (1.3)	8.5 (2.1)	12.7 (1.8)
*r_c_^e^*(nm)	29.2 (0.6)	27.0 (0.6)	19.6 (0.5)	19.8 (0.5)	7.3 (1.8)	8.3 (0.6)	8.5 (2.1)	12.7 (1.8)
*t_f__,c_^f^*(nm)	5.5	6.6	2.4	3.5	1.5	2.6	1.0	1.6
*t_s_^g^*(nm)	14.0 (0.8)	16.2 (0.5)	10.0 (0.6)	10.0 (0.9)	7.2 (1.8)	8.2 (1.1)		
*t_f__,s_^h^*(nm)	1.5	1.5	2.6	1.5	1.7	1.5		
*ε ^i^*	1.0	1.0	1.0	1.0	1.0	1.0	1.0	1.0
*ξ ^j^* (nm)	4.6	5.2	5.4	4.7	5.0	2.9		
*R*_g_*^k^* (nm)	33.5	33.5	22.9	23.1	11.2	12.9	6.6	9.8
*R*_g,IFT_*^l^* (nm)	33.8	33.7	23.2	23.3	12.9	13.5	8.9	10.4
*R*_max,IFT_*^m^* (nm)	45.5	45.1	30.9	31.2	16.3	18.0	10.7	14.1
*D*_max,IFT_*^n^* (nm)	87.5	86.8	63.0	62.4	38.0	38.4	25.4	30.0
*R*_max,IFT_ */R*_g,IFT_	1.35	1.34	1.33	1.34	1.26	1.33	1.20	1.36
*D_max_/R*_max,IFT_	1.92	1.92	2.04	2.00	2.33	2.13	2.37	2.13
*R*_g_ */R*_g,IFT_	0.99	0.99	0.99	0.99	0.87	0.96	0.74	0.94
*DLS Analysis*								
*R*_h,z_*^o^* (nm)	33.5		25.0		14.0		10.4	
*PDI*_DLS_*^p^* (nm)	0.154		0.055		0.085		0.093	
*R*_h,intensity_*^q^* (nm)	39.9 (14.7)		26.9 (7.4)		15.3 (4.7)		11.6 (3.9)	
*R*_h,voulme_*^r^* (nm)	28.0 (10.5)		21.9 (6.3)		11.9 (3.7)		8.6 (2.9)	
*R*_h,number_*^s^* (nm)	21.6 (6.3)		18.6 (4.5)		9.9 (2.5)		7.0 (1.9)	
*R*_h,z_/*R*_g_	1.00	1.00	1.09	1.08	1.25	1.09	1.58	1.06
*R*_h,z_/*R*_g,IFT_	0.99	0.99	1.08	1.07	1.09	1.04	1.17	1.00
*R*_h,intensity_/*R_e_*	0.92	0.92	0.91	0.90	1.06	0.93	1.36	0.91
*R*_h,volume_/*R_e_*	0.65	0.65	0.74	0.73	0.82	0.72	1.01	0.68
*R*_g,number_/*R_e_*	0.50	0.50	0.63	0.62	0.68	0.60	0.82	0.55
*TEM Analysis*								
*R ^t^* (nm)	40.5 (1.5)		25.5 (1.5)					

*^a^*The scattering from water itself was measured and employed in the data analysis. *^b^*The scattering from the supernatant was measured and employed in the data analysis. *^c^*Radius of particle in equatorial direction. *^d^*Standard deviation. *^e^*Radius of particle core. *^f^*Thickness of the fuzzy part (interfaced with the shell) of the core. *^g^*Thickness of shell part. *^h^*Thickness of the fuzzy part (interfaced with water or supernatant) of the shell.*^i^*Ellipsoidicity ratio (polar radius/equatorial radius).*^j^*Average correlation length of density fluctuation (i.e., random two phases) in the whole particle. *^k^*Radius of gyration of particle. *^l^*Radius of gyration determined from IFT analysis. *^m^*Radius determined from the peak maximum of the *p*(*r*) function in IFT analysis. *^n^*Maximum dimension determined from the *p*(*r*) function in IFT analysis.*^o^*z-Averaged hydrodynamic radius. *^p^*Polydispersity index of hydrodynamic radius. *^q^*Intensity-weighted mean radius. *^r^*Volume-weighted mean radius. *^s^*Number-weighted mean radius. *^t^*Mean radius of particle determined by transmission electron microscopy (TEM) analysis (data from [61]).

## Supplementary Materials

The following are available online at https://www.mdpi.com/2073-4360/12/2/477/s1, Figure S1: Pair distance distribution functions *p*(*r*) obtained from numerical Fourier transformation of the extrapolated scattering intensity profile from model analysis, Table S1: Structural parameters of PS nanoparticles determined from *p(r)* function obtained by numerical Fourier transformation of respective extrapolated scattering intensity profile acquired in the quantitative structural model analysis.
