# Supplementary material for: Quantitative Structural Analysis of Polystyrene Nanoparticles Using Synchrotron X-ray Scattering and Dynamic Light Scattering
Source: Polymers (Basel). 2020 Feb 19;12(2):477. doi: 10.3390/polym12020477 (PMC7077714; doi:10.3390/polym12020477)
Supplement: Supplementary file 1 [file polymers-12-00477-s001.pdf]

Supporting Information

Quantitative Structural Analysis of Polystyrene Nanoparticles Using Synchrotron X-Ray Scattering and Dynamic Light Scattering

Jia Chyi Wong [1,2], Li Xiang [2], Kuan Hoon Ngoi [1,2], Chin Hua Chia [1,*], Kyeong Sik Jin [3,*] and Moonhor Ree [2,*]

[1] Materials Science Program, School of Applied Physics, Faculty of Science and Technology, Universiti Kebangsaan Malaysia, 43600 Bangi, Selangor, Malaysia; wongjiachyi@gmail.com (J.C.W.); ngoikuanhoon@gmail.com (K.H.N.)
[2] Department of Chemistry, Polymer Research Institute, and Pohang Accelerator Laboratory, Pohang University of Science and Technology, Pohang 37673, Republic of Korea; lea1990@postech.ac.kr
[3] Pohang Accelerator Laboratory, Pohang University of Science & Technology, Pohang 37673, Republic of Korea
* Correspondence: chia@ukm.edu.my (C.H.C); jinks@postech.ac.kr (K.S.J.); ree@postech.ac.kr (M.R.)

1. X-ray Scattering Data Analysis

The scattering intensity $I(q)$ from particles in a solution can be generally analyzed by two different approaches, namely structural model approach and model-independent indirect Fourier transformation (IFT) method [1-3].

First, as a structural model approach, the scattering intensity $I(q)$ of particles in a solution can be expressed by the following equation [1-5]:

$$I(q) = n_p \, P(q) \, S(q) \qquad (1)$$

where $n_p = N_p/V$ is the number density of particles in the sample where N_p is the number of particles and V is the solution sample volume exposed to the X-ray beam employed, $P(q)$ is the form factor of the particle, and $S(q)$ is the structure factor for the particles. The intensity is measured as a function of scattering vector $q = (4\pi \sin \theta)/ \lambda$, where 2θ is the scattering angle and λ is the wavelength of the X-ray beam.

In this study, X-ray scattering measurements were conducted for diluted particle suspensions and, therefore, the $S(q)$ term can be approximated to be unity over the entire q range [1-3,6]:

$$S(q) \approx 1. \quad (2)$$

This approximation allows the isolation of $P(q)$ from the overall scattering intensity $I(q)$. Therefore, equation 1 can be written as:

$$I(q) = n_p P(q). \quad (3)$$

The core-shell ellipsoid model has been developed to account colloidal particles that are not perfectly sphere in shape [1-3,7]; the geometry of such spheroid particle can be defined by the ellipticity (ε), namely the ratio between polar axis (R_p) and equatorial axes (R_e) as below:

$$\varepsilon = \frac{R_p}{R_e} \quad (4)$$

where prolate ellipsoid is obtained when $R_e > R_p$ (i.e., $\varepsilon < 1$) and oblate ellipsoid is obtained when $R_e < R_p$ (i.e., $\varepsilon > 1$). In the solution x-ray scattering measurement, the ellipsoidal of revolution is considered by convolution of spherical scattering amplitudes (Φ) with Gaussian smoothing function to account the region of fuzzy interface ($2\sigma_f$) with gradual decrease of radial scattering length density distribution [1-3,7,8]. The resulting scattering amplitude is expressed as following:

$$\Phi\left(q, \sigma_f, r'(\alpha, R_e)\right) = \frac{3\left(\sin(qr') - qr'\cos(qr')\right)}{(qr')^3} \,.\exp\left(-\frac{(q\sigma_f)^2}{4}\right) \quad (5)$$

where r' is the effective radii of R_e and R_p in radians of $0 \leq \alpha \leq \pi/2$, which can be expressed by

$$r'(\alpha, R_e) = R_e \sqrt{\sin^2\alpha + \varepsilon^2 \cos^2\alpha} \,. \quad (6)$$

Additionally, the core-shell ellipsoidal particle is considered to account different phases in single particle. The total scattering amplitude (A_{total}) is expanded into scattering amplitude of core (A_{core}) and scattering amplitude of shell (A_{shell}) as below [3,9]:

$$A_{core}\left(q, r'\left(\alpha, R_{e,\ core}\right)\right) = \Delta\rho_{core}(r')\ V_{core}(R_{e,\ core})\ \Phi_{core}\left(q, \sigma_{f,\ core}, r'\left(\alpha, R_{e,\ core}\right)\right) \tag{7}$$

$$A_{shell}\left(q,\ r'\left(\alpha, R_{e,\ core}\right)\right) = \Delta\rho_{shell}(r')V_{shell}(R_{e,shell})\ \Phi_{shell}\left(q, \sigma_{f,\ shell}, r'\left(\alpha, R_{e,\ shell}\right)\right) \tag{8}$$

$$A_{total} = A_{core}\left(q,\ r'\left(\alpha, R_{e,\ core}\right)\right) + A_{shell}\left(q,\ r'\left(\alpha, R_{e,\ core}\right)\right) \tag{9}$$

where volume of ellipsoid is described as below:

$$V(R_e) = \frac{4\pi}{3}\varepsilon R_e^3\ . \tag{10}$$

Subsequently, the form factor of ellipsoid of revolution (P_e) can be obtained by orientational averaging of the form factor P as below:

$$P\left(q, r'(\alpha, R_e)\right) = A_{total}\left(q, r'(\alpha, R_e)\right)^2 \tag{11}$$

$$P_e(q, R_e) = \int_0^{\frac{\pi}{2}} P(q, r'(\alpha, R_e)) \sin\alpha\, d\alpha. \tag{12}$$

Moreover, the radial scattering length density profile $\Delta\rho(r)$ along equatorial axis R_e of a single particle can be further obtained by numerical Fourier transformation of the respective scattering amplitudes:

$$\Delta\rho(r) = \frac{1}{2\pi^2}\int A\left(q, r'(\alpha, R_e)\right)\frac{\sin(qr)}{qr}dq \tag{13}$$

where $\alpha = \frac{\pi}{2}$ and $r'(\alpha, R_e) = R_e$.

In this study, the model generated scattering amplitude is extrapolated to the range of $2 < q \leq 35$ nm^{-1} from the measurable range of $0.07 \leq q \leq 2$ nm^{-1} to avoid termination ripples due to the

insufficient experimental range of q. The extrapolated range of q lower than 0.007 nm^{-1} contributes insignificantly on the transformed $\Delta\rho(r)$.

To consider polydisperse particles, the Schultz-Zimm distribution function is assumed [4]:

$$n(R_e) = \frac{\left(\frac{z+1}{R_0}\right) R_e^{\,z}}{\Gamma(z+1)} \exp\left(\frac{-(z+1)R_e}{R_0}\right) \tag{14}$$

$$z = \left(\frac{R_0}{\sigma}\right)^2 - 1 \tag{15}$$

where z, R_0 and σ are the width parameter, mean of the size distribution and root mean square deviation from R_0, respectively. $\Gamma(x)$ is the Gamma function. The Gaussian distribution function is approximated at $z \approx 0$ and right-skewed distribution function is approximated at $z \gg 1$. After incorporating all contributions, including constant incoherent background $\left(I_{bckg}\right)$ [8], the number-averaged intensity distribution is expressed as below:

$$I(q, R_e) = n_p \int_0^{\frac{\pi}{2}} \int_0^{\infty} n(R_e)\, P\left(q, r'(\alpha, R_e)\right) \mathrm{d}R_e \sin\alpha\, \mathrm{d}\alpha + I_{bckg}. \tag{16}$$

To consider X-ray scattering intensities from possible inhomogeneities inside a single particle, structure, an additional form factor $P_a(q)$ can be expressed as the function of correlation length l_p which is assumed to be originated from random two phases [10]:

$$P(q) = \int_0^{\infty} \mathrm{e}^{-\frac{r}{l_p}} \frac{\sin(qr)}{qr} r^2 dr. \tag{17}$$

On the other hand, the structure factor $S(q)$ from the particles system can be given as below [5,11,12]:

$$S(q) = \frac{1}{1 - N(q)} \tag{18}$$

where

$$N(q) = -24\phi\left(\begin{array}{c}\lambda_1\frac{\sin D - D\sin D}{D^3} - 6\phi\lambda_2\frac{D^2\cos D - 2D\sin D - 2\cos D + 2}{D^4} \\ -\phi\lambda_1\frac{D^4\cos D - 4D^3\sin D - 12D^2\cos D + 24D\cos D - 24}{2D^6}\end{array}\right) \quad (19)$$

$$\lambda_1 = \frac{(1+2\phi)^2}{(1-\phi)^4} \quad (20)$$

$$\lambda_2 = \frac{(1+\frac{\phi}{2})^2}{(1-\phi)^4}. \quad (21)$$

Here, ϕ is the volume fraction of minor phase domain, and $D = 2l_D q$ where l_D indicates the size of the phase separated domain and it is assumed that l_D is equal to l_p which is the correlation length used in the form factor for simplicity in consideration of randomly phase separated structure model.

As a second approach, the IFT method is applicable to analyze the scattering intensity $I(q)$ from particles in a solution [1-3,7,11,13-16]. With the priori information of maximum dimension, D_{max}, the radius of gyration, R_g and radius at peak maximum, R_{max} are obtained from the pair distance distribution function $p(r)$ by assuming the $p(r)$ as the series below:

$$p(r) = \sum_{i=1}^{N} c_i \ \varphi_i(r) \qquad \text{for } 0 \le r \le D_{max} \quad (22)$$

where N is the number of functions, c_i are the unknown expansion coefficients and $\varphi_i(r)$ are the cubic B splines.

Due to the linearity of Fourier transformation,

$$I(q) = \sum_{i=1}^{N} c_i \ \psi_i(q) \quad (23)$$

where $I(q)$ is the Fourier transform of $p(r)$ and is represented by the series of Fourier transformed B splines $\psi_i(q)$.

Under the constraint of N_c,

$$N_c = \sum_{i=1}^{N-1} (c_{i+1} - c_i)^2 \quad (24)$$

together with the Lagrange multiplier, λ under least square condition,

$$(\mathrm{L}+\lambda\cdot\mathrm{N_c}) = \mathrm{Min} \tag{25}$$

c_i is then calculated by weighted least-squares operation:

$$\mathrm{L} = \sum_{k=1}^{M} \frac{[I_{\exp}(q_k)\text{-}I_{\exp}(q_k)]^2}{\sigma^2(q_k)} = \sum_{k=1}^{M} \frac{[I_{\exp}(q_k)\text{-}\sum_{i=1}^{N} c_i\,\psi_i(q_k)]^2}{\sigma^2(q_k)} \tag{26}$$

Thus, the $p(r)$ profile is then obtained with the optimized c_i indirectly.

Alternatively, the $p(r)$ can also be obtained by numerical Fourier transformation of the scattering intensity profile $I(q)$ which is reconstructed with the structural parameter details determined from the structural model analysis of the experimentally measured scattering data (Figure S1). This approach requires good matches between the measured $I(q)$ and the model generated $I(q)$. In this study, the model generated $I(q)$ is extrapolated to the range of $0.001 \leq q < 0.07$ nm^{-1} from the measurable range of $0.07 \leq q \leq 2$ nm^{-1} to avoid termination ripples due to the insufficient experimental range of q. The extrapolated range of q higher than 2 nm^{-1} contributes insignificantly to the transformed $p(r)$. The $p(r)$ is obtained with the equation below:

$$p(r) = \frac{1}{2\pi}\int I(q)\, qr \sin(qr)\, dq. \tag{27}$$

From the $p(r)$ function profile, the radius of gyration (R_g) of the particle can be estimated by using the following relation:

$$R_g{}^2 = \frac{\int p(r)\ r^2\,\mathrm{d}r}{2\int p(r)\,\mathrm{d}r}\ . \tag{28}$$

Moreover, the radius distributions (i.e., number-weighted radius distribution: $n(R_e)$ versus R_e), which are determined from the scattering data by the quantitative structural model analysis, can be further converted to the scattering intensity-weighted radius distribution by using the following relation [17]:

$$I(q) = c_n \int_0^{\infty} n(R_e) \cdot R_e^6 \cdot I_1(qR_e) dR_e \qquad (29)$$

where $I_1(qR_e)$ is the scattered intensity from a particle of radius R_e and c_n is a constant.

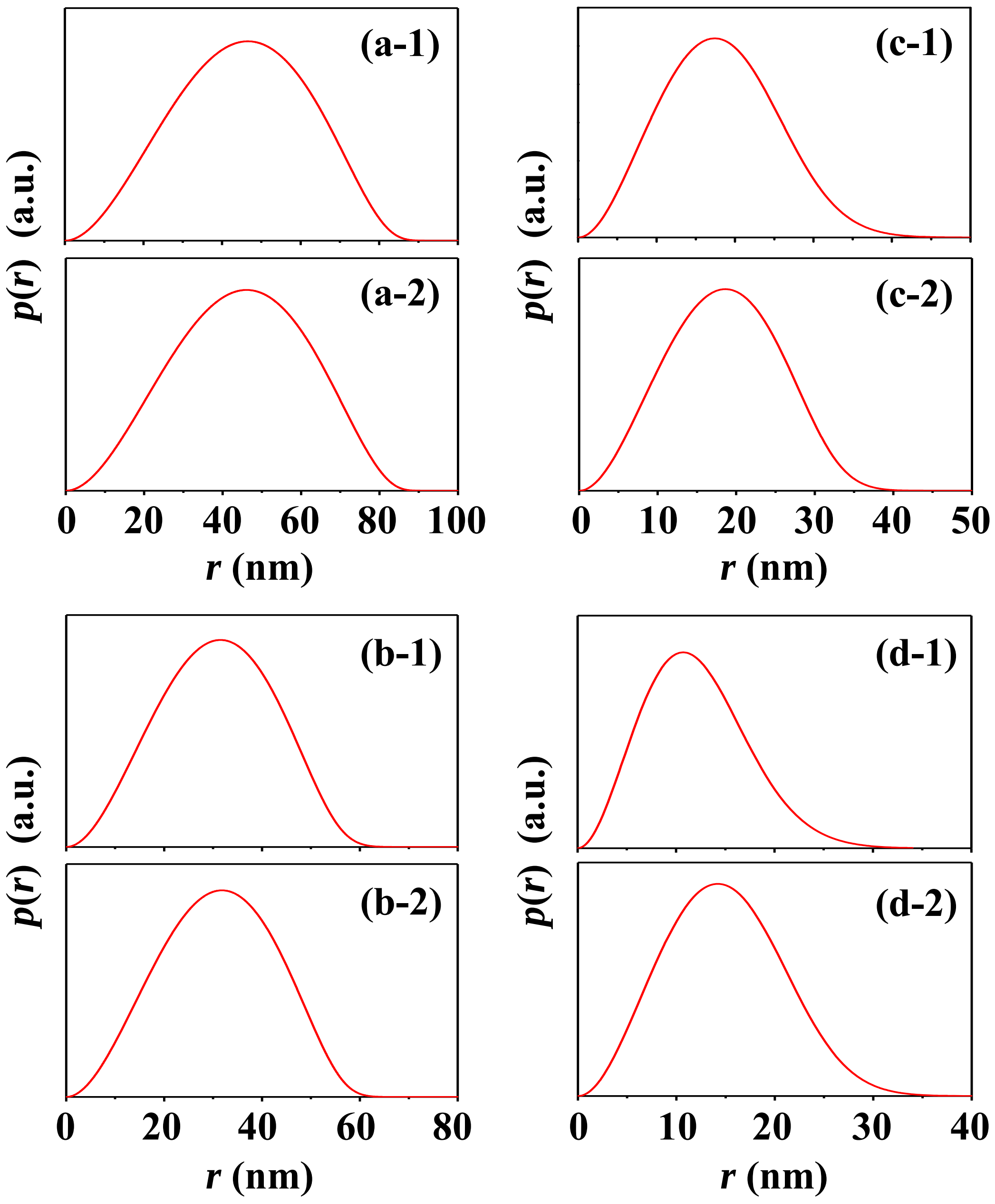

Figure S1. Pair distance distribution functions $p(r)$ obtained from numerical Fourier transformation of the extrapolated scattering intensity profile from model analysis: (a-1) PS-1, corrected with water as the solution medium; (a-2) PS-1, corrected with supernatant as the solution medium; (b-1) PS-2, corrected with water as the solution medium; (b-2) PS-2, corrected with supernatant as the solution medium; (c-1) PS-3, corrected with water as the solution medium; (c-2) PS-3, corrected with

supernatant as the solution medium; (d-1) PS-4, corrected with water as the solution medium; (d-2) PS-4, corrected with supernatant as the solution medium.

Table S1. Structural parameters of PS nanoparticles determined from *p(r)* function obtained by numerical Fourier transformation of respective extrapolated scattering intensity profile acquired in the quantitative structural model analysis.

Structure parameter	PS Nanoparticles							
	PS-1		PS-2		PS-3		PS-4	
	Water	Supernatant	Water	Supernatant	Water	Supernatant	Water	Supernatant
SAXS Analysis								
R_g [a] (nm)	34.0	33.8	23.4	23.5	13.6	13.5	9.3	11.3
R_{max} [b] (nm)	46.6	46.1	31.5	31.8	16.6	18.0	10.7	14.2
D_{max} [c] (nm)	87.5	87.0	63.0	62.5	44.0	38.7	33.1	37.5
R_{max}/R_g	1.37	1.36	1.35	1.35	1.22	1.33	1.15	1.26
D_{max}/R_{max}	1.88	1.88	2.00	1.96	2.65	2.15	3.09	2.64

[a]Radius of gyration. [b]Radius at peak maximum. [c]maximum dimension. [d]Radius of gyration of particle from the model analysis.

2. DLS Data Analysis

In a homogeneous dispersion, the particles scatter the incident light into all directions. They move in three-dimensional random-walk motion under diffusion process. At a fixed scattering angle, the scattered intensity shows stochastic fluctuations due to their time-dependent positions and momenta. This fluctuating signal about its mean intensity is collected by using a detector for a duration of time and then the normalized autocorrelation function of scattered intensity, $g_2(q, t)$ can be obtained as below [1,18-21]:

$$g_2(q, t) = \frac{\langle I_s(q, t) I_s(q, t+\tau)\rangle}{\langle |I_s(q, t)|^2\rangle} \tag{30}$$

where t is the time, τ is the delayed time, and q is the magnitude of scattering vector defined by the equation below:

$$q = \frac{4\pi n_o \sin\theta}{\lambda} \tag{31}$$

where n_o is the refractive index of a solvent used, λ is the wavelength of a laser light used, and 2θ is the scattering angle. When τ is approaching zero, $I(q, t+\tau)$ is correlated with $I_s(q, t)$ and this correlation disappears at large τ. Thus, the $g_2(q, t)$ of a nonperiodic property decay from its initial

value of $\langle I_s^2 \rangle$ to a final value of $\langle I_s \rangle^2$. The Siegert relation [22] describes the $g_2(q, t)$ by normalized scattered field equation of $g_1(q, t)$ as below:

$$g_2(q, \tau) = 1 + \beta \ |g_1(q, t)|^2 \tag{32}$$

with β is a instrumental optical corrections. For a monodisperse and dilute dispersion of particles,

$$g_1(q, t) = \exp(-\Gamma\tau) \tag{33}$$

where $\Gamma = q^2 D_t$ is the decay rate constant and related to the translational diffusion coefficient D_t of the particles. In the case of spherical particles, the hydrodynamic radius, R_h is expressed by the Stokes-Einstein equation [23,24] as below:

$$R_h = \frac{k_B T}{6\pi\eta D_t} \tag{34}$$

where k_B is the Boltzmann constant, T is the temperature of sample, and η is the (temperature-dependent) viscosity of the dispersant. For the polydisperse dispersion, $g_1(q, \tau)$ is the integral over exponential terms weighted by $G(\Gamma)$, or a continuous distribution of decay rate constants Γ as below [19,25]:

$$g_1(q, \tau) = \int G(\Gamma)\exp(-\Gamma\tau)d\Gamma. \tag{35}$$

For a narrow monomodal distribution of $G(\Gamma)$, the Cumulant method [26,27] is generally used to analyze the deviation from linearity of equation below:

$$\ln\left(g_1(q, \tau)\right) = \sum_1^\infty k_m(\Gamma)\frac{(-\tau^m)}{m!} \tag{36}$$

where $k_m(\Gamma)$ is the mth cumulant values, which can be obtained from the curve of $\ln\left(g_1(q, \tau)\right)$ versus τ. $k_1(\Gamma)$ is the mean of $G(\Gamma)$ or z-averaged translational diffusion coefficient D_z of particles:

$$k_1(\Gamma) = \overline{\Gamma} = \int G(\Gamma)\,\Gamma \mathrm{d}\Gamma = q^2 D_z. \tag{37}$$

From D_z, the hydrodynamic radius R_h of the particle (specially, *hard spherical particle*) can be calculated by using the Stokes-Einstein equation [24,28] and the viscosity of the surrounding medium:

$$R_{h,z} = \frac{k_B T}{6\pi\eta D_z}. \quad (38)$$

Together with $\overline{\Gamma}$ and $k_2(\Gamma)$ or the variance of $G(\Gamma)$, the polydispersity index (PDI_{DLS}) is calculated as below:

$$PDI_{DLS} = \frac{k_2(\Gamma)}{\overline{\Gamma}^2}. \quad (39)$$

Since the Cumulant method is less descriptive for samples that have size distribution further away from unimodal, the non-negatively constrained least square (NNLS) deconvolution algorithm can be applicable to analyze the DLS data from samples with larger polydispersity, one common example is the CONTIN algorithms [29,30].

For small and isotropic spherical particles, the scattering intensity is proportional to the radius to the sixth power. Thus, in terms of scattering intensity, the relative contribution from the spherical particles with a radius a can be expressed as follows [31]:

$$\text{Intensity (\%)} \ [I_a(\%)] = \frac{100 \cdot N_a a^6}{N_a a^6 + N_b b^6 + \cdots} \ . \quad (40)$$

For the spherical particles, the particle volume is proportional to the radius to the third power. Taking this into account, in terms of volume, the relative contribution from the particles with a radius a is

$$\text{Volume (\%)} \ [V_a(\%)] = \frac{100 \cdot N_a a^3}{N_a a^3 + N_b b^3 + \cdots} \ . \quad (41)$$

Moreover, in terms of particle in number, the relative contribution from the particles with a radius a is

$$\text{Number(\%)} \ [N_a(\%)] = \frac{100 \cdot N_a}{N_a + N_b + \cdots} \ . \quad (42)$$

References

1. Ree, B. J.; Lee, J.; Satoh, Y.; Kwon, K.; Isono, T.; Satoh, T.; Ree, M. A comparative study of dynamic light and X-ray scatterings on micelles of topological polymer amphiphiles. *Polymers* **2018**, *10*, 1347.
2. Ree, B. J.; Satoh, T.; Yamamoto, T. Micelle structure details and stabilities of cyclic block copolymer amphiphile and its linear analogues. *Polymers* **2019**, *11*, 163.
3. Ree, B. J.; Satoh, Y.; Jin, K. S.; Isono, T.; Kim, W. J.; Kakuchi, T.; Satoh, T.; Ree, M. Well-defined and stable nanomicelles self-assembled from brush cyclic and tadpole copolymer amphiphiles: a versatile smart carrier platform. *NPG Asia Materials* **2017**, *9*, e453.
4. Li, T.; Senesi, A. J.; Lee, B. Small angle X-ray scattering for nanoparticle research. *Chem. Rev.* **2016**, *116*, 11128-11180.
5. Pedersen, J. S. Analysis of small-angle scattering data from colloids and polymer solutions: modeling and least-squares fitting. *Adv. Colloid Interf. Sci.***1997**, *70*, 171-210.
6. Kotlarchyk, M.; Chen, S. H. Analysis of small angle neutron scattering spectra from polydisperse interacting colloids. *J. Chem. Phys.* **1983**, *79*, 2461-2469.
7. Rathgeber, S.; Monkenbusch, M.; Kreitschmann, M.; Urban, V.; Brulet, A. Dynamics of star-burst dendrimers in solution in relation to their structural properties. *J, Chem. Phys.* **2002**, *117*, 4047-4062.
8. Stieger, M.; Richtering, W.; Pedersen, J. S.; Lindner, P. Small-angle neutron scattering study of structural changes in temperature sensitive microgel colloids. *J. Chem. Phys.* **2004**, *120*, 6197-6206.
9. Schmid, A. J.; Dubbert, J.; Rudov, A. A.; Pedersen, J. S.; Lindner, P.; Karg, M.; Potemkin, I. I.; Richtering, W. Multi-shell hollow nanogels with responsive shell permeability. *Scientific Rep.* **2016**, *6*, 22736.
10. Debye, P.; Bueche, A. M. Scattering by an inhomogeneous solid. *J. Appl. Phys.* **1949**, *20*, 518-525.
11. Roe, R. J. *Methods of X-ray and neutron scattering in polymer science*. Vol. 739, Oxford Univ. Press: New York, 2000.
12. Ahn, B.; Kim, D. M.; Hsu, J.-C.; Ko, Y.-G.; Shin, T. J.; Kim, J.; Chen, W.-C.; Ree, M. Tunable film morphologies of brush–linear diblock copolymer bearing difluorene moieties yield a variety of digital memory properties. *ACS Macro Lett.* **2013**, *2*, 555-560.
13. Glatter, O. A new method for the evaluation of small-angle scattering data. *J. Appl. Cryst.* **1977,** *10*, 415-421.

14. Müller, K.; Glatter, O. Practical aspects to the use of indirect Fourier transformation methods. *Makromol. Chem.* **1982**, *183*, 465-479.

15. Glatter, O.; Hainisch, B. Improvements in real-space deconvolution of small-angle scattering data. *J. Appl. Cryst.* **1984,** *17*, 435-441.

16. Mittelbach, R.; Glatter, O. Direct structure analysis of small-angle scattering data from polydisperse colloidal particles. *J. Appl. Cryst.* **1998,** *31*, 600-608.

17. Glatter, O. *Scattering Methods and their Application in Colloid and Interface Science*. Elsevier: Amsterdam, 2018.

18. Percus, J. K.; Yevick, G. J. Analysis of classical statistical mechanics by means of collective coordinates. *Phys. Rev.* **1958**, *110*, 1.

19. Berne, B. J.; Pecora, R. *Dynamic Light Scattering: with Applications to Chemistry, Biology, and Physics*. Dover Publications: Mineola, New York, 2000.

20. Stetefeld, J.; McKenna, S. A.; Patel, T. R. Dynamic light scattering: A practical guide and applications in biomedical sciences. *Biophys. Rev.* **2016**, *8*, 409-427.

21. Pecora, R. Dynamic light scattering measurement of nanometer particles in liquids. *J. Nanoparticle Res.* **2000**, *2*, 123-131.

22. Siegert, A. *On the fluctuations in signals returned by many independently moving scatterers*. Radiation Laboratory, Massachusetts Institute of Technology, 1943.

23. Einstein, A. Über die von der molekularkinetischen Theorie der Wärme geforderte Bewegung von in ruhenden Flüssigkeiten suspendierten Teilchen. *Annalen der Physik* **1905**, *322*, 549-560.

24. Einstein, A. Zur theorie der brownschen bewegung. *Annalen der Physik* **1906**, *324*, 371-381.

25. Aragon, S.; Pecora, R. Theory of dynamic light scattering from polydisperse systems. *J. Chem. Phys.* **1976**, *64*, 2395-2404.

26. Koppel, D. E. Analysis of macromolecular polydispersity in intensity correlation spectroscopy: the method of cumulants. *J. Chem. Phys.* **1972**, *57*, 4814-4820.

27. Hassan, P.; Kulshreshtha, S. Modification to the cumulant analysis of polydispersity in quasielastic light scattering data. *J. Colloid Interf. Sci.* **2006**, *300*, 744-748.

28. Guinier, A.; Fournet, G. *Small Angle Scattering X-Ray*; Wiley: New York, NY, USA, 1955.

29. Provencher, S. W. A constrained regularization method for inverting data represented by linear algebraic or integral equations. *Computer Phys. Comm.* **1982**, *27*, 213-227.

30. Provencher, S. W. CONTIN. A general purpose constrained regularization program for inverting noisy linear algebraic and integral equations. *Computer Phys. Comm.* **1982**, *27*, 229-242.

31. https://www.malvernpanalytical.com/en/learn/knowledge-center/technical-notes/TN101104IntensityVolumeNumber.html
